# Metal Nanoparticles’ Biological Activity and Pharmaceutical Applications

**DOI:** 10.3390/ph18101537

**Published:** 2025-10-13

**Authors:** Elitsa L. Pavlova, Debbie C. Crans

**Affiliations:** 1 Optics and Spectroscopy Department, Faculty of Physics, Sofia University “St. Kliment Ohridski”, 5 James Bourchier Blvd., 1164 Sofia, Bulgaria; 2 Department of Chemistry and Cell and Molecule Biology Program, Colorado State University, Fort Collins, CO 80532, USA; debbie.crans@colostate.edu

Because of their variable physicochemical characteristics, metal nanoparticles are suitable and highly favorable for introduction to, and use in, living systems. The latest research on the design, synthesis, properties, biological evaluation, and pharmaceutical application of various metal nanoparticles is presented in the form of three reviews and nine original research articles in this Special Issue. The contributions highlight the importance of designed nanoparticle synthesis, including “green” and bio-inspired methods, as well as multiple approaches to enhancing their efficacy and safety through physicochemical and biological means [1,2,3]. The applications of metal nanoparticles in biology and medicine are described in articles ranging from toxicity studies to studies on antimicrobial activity against multiple Gram-positive and Gram-negative bacteria [4,5,6,7], diabetes [8], and several types of cancers [9,10], although many challenges with regard to design, toxicity, specificity, and cellular uptake remain and are addressed herein [9,11,12,13,14,15,16,17,18,19,20]. Nanoparticles prepared from silver, gold, manganese oxides, copper oxides, zinc oxides, zeolites, rare earth metals, and small polyoxovanadates, as well as nanoparticles laced with other metals or biomaterials, are described, along with their particular application and mechanisms of action. The properties of the nanoparticles depend strongly on their shape, size, and material, as well as the load they carry. The ability of metal nanoparticles to interact with cells, as well as their structures and cellular metabolites, is of particular importance, as highlighted in their applications as drug carriers and in the diagnosis and treatment of various diseases. Such representable examples are described in this Special Issue.

“Green” and bio-inspired syntheses, recently developed, address concerns about eco-safety and toxicity while enhancing the bioactivity of the newly composed metal nanoparticles [2,3]. Dolashka et al. [Contribution 1] utilized the mucus of the garden snail, *Cornu aspersum*, as a natural reducing and stabilizing agent to produce copper oxide nanoparticles. The newly synthesized nanoparticles were spherical, ~150 nm, and demonstrated superior antimicrobial activity against multiple Gram-positive (*Staphylococcus*) and Gram-negative bacteria (*Escherichia coli* and *Salmonella*) when compared to the mucus alone. Importantly, proteins present in the snail mucus contributed to nanoparticle stabilization and enhanced their bactericidal efficacy.

Alsaleh et al. [Contribution 2] synthesized zinc oxide-doped manganese oxide nanoparticles with improved catalytic properties and demonstrated their relatively low toxicity in murine macrophages. The doping altered nanoparticle uptake and increased the generation of reactive oxygen species and the activation of the cell antioxidant system. The obtained findings suggest that ZnO (1%) doping impacts cellular bio-interaction and the consequent toxicological outcomes.

Asif and colleagues [Contribution 3] investigated enhanced doping methods by synthesizing aluminum-doped zinc oxide nanocomposites. Notably, the Zn_0.75_Al_0.25_O formulation demonstrated the most pronounced antimicrobial efficacy, particularly targeting *Bacillus cereus*. In addition, these nanocomposites exhibited selective cytotoxicity against liver, breast, and ovarian cancer cell lines. Importantly, their minimal toxicity toward normal liver cells suggests potential for safer anticancer applications.

The antimicrobial properties of silver-based nanomaterials have been the subject of recent investigations [4,5,6,7]. In a study by Pavlova et al. [Contribution 4], the researchers investigated silver-exchanged zeolites with varied silver content and Si/Al ratios. Their findings demonstrated a clear proportional relationship between the release of active silver species and the materials’ antibacterial and bactericidal efficacy. In general, as the zeolites released more silver, their effectiveness against bacterial pathogens increased. However, the leaching of silver ions also raised important environmental concerns. The bactericidal concentrations of silver ions also caused significant mortality in *Daphnia magna*—a commonly used bioindicator in ecotoxicology. This result underscores the challenge of balancing antimicrobial efficacy with environmental safety, highlighting the potential ecological risks associated with spreading silver-based materials capable of producing effective antimicrobial concentrations.

Stoyanova and colleagues [Contribution 5] developed silver nanoparticles encapsulating mebeverine, an antispasmodic agent commonly used for irritable bowel syndrome, along with a structural analogue. Their in vitro studies indicated that cytotoxicity was concentration-dependent, but importantly, no genotoxic effects were observed at the concentrations investigated. These findings suggest that there is a high potential for the nanoparticle-based delivery system to be both safe and effective for potential therapeutic applications.

Rehman and colleagues [Contribution 6] utilized *Azadirachta indica* seed extract to biosynthesize silver nanoparticles exhibiting notable antidiabetic properties. In vitro analyses revealed that these nanoparticles significantly enhanced glucose uptake and adsorption and more effectively inhibited α-amylase activity compared to crude extracts. Extending their research in vivo, the team observed that diabetic mice treated with these nanoparticles experienced marked hypoglycemic effects and tissue regeneration in both the pancreas and liver. In general, these findings highlight the considerable therapeutic potential of “green”-synthesized silver nanoparticles derived from *Azadirachta indica*.

Originally, Rangel-López et al. [Contribution 7] introduced an innovative approach to synthesize gold nanoparticles, employing triple-negative breast cancer cell lysate as a reducing agent with immunomodulatory properties. This method resulted in nanoparticles that enhanced antigen presentation and stimulated proinflammatory cytokine secretion, while also increasing populations of CD8+ and CD22+ lymphocytes in murine models. These immunological effects translated into improved survival rates and inhibited tumor implantation, highlighting the nanoparticles’ significant potential in advancing cancer immunotherapy strategies, in agreement with the suggestions made previously [9,10].

In another investigation, Gawas et al. [Contribution 8] successfully synthesized gold nanoparticles utilizing *Cordyceps militaris*, a medicinal mushroom, by a green, quality-by-design method. The obtained nanoparticles exhibited significant stability and biocompatibility in addition to pronounced antioxidant, antidiabetic, and antibacterial properties. FTIR spectroscopy analysis revealed that bioactive constituents derived from the mushroom played a pivotal role in the stabilization of the nanoparticles, thereby emphasizing the environmentally sustainable characteristics and multifunctional potential of these materials for prospective biomedical applications.

Panchal et al. [Contribution 9] conducted a comprehensive review focusing on the biomedical applications of plant-mediated silver, gold, and zinc oxide nanoparticles. Plant-based synthesis of metal-based nanoparticles utilizes natural extracts as reducing and stabilizing agents and, in this manner, minimize harmful leaching and other toxic by-products. They focused on their antioxidant, antimicrobial, and anticancer activities. The review emphasized the advantages of the “green” synthetic approaches using plant-based materials, which, in turn, result in enhanced therapeutic activity and improved safety compared to reported conventional synthetic approaches. Furthermore, the authors discussed the potential integration of metal nanoparticles with graphitic carbon nitride to create hybrid nanocomposites. Such materials could open new applications in drug delivery, diagnostics, and environmental remediation.

Burlec et al. [Contribution 10] comprehensively reviewed the synthesis, characterization, and therapeutic applications of mainly silver and gold nanoparticles. Their paper outlined the roles of nanoparticles in drug delivery, antimicrobial activity, cancer therapy, and diagnostic procedures, including data from over thirty clinical trials. Despite these promising findings, the authors direct the readers’ attention to ongoing challenges related to biocompatibility and regulatory approval, which is delaying the approval for the use of these approaches and explains why so few of these metal nanoparticle systems are used clinically. As a result, this paper highlights the fact that challenges are not only scientific but also include legal limitations before metal nanoparticles technologies achieve broader clinical applications.

Barbosa and colleagues [Contribution 11] carried out an in-depth toxicity evaluation of a small, nanosized, mixed-valence polyoxovanadate cluster, employing both in vitro models (peripheral blood mononuclear cells and *Artemia salina*) and *in vivo* studies (mice). This cluster is unique because it is redox-active and hence differs from other polyoxometalate clusters. The study showed that it demonstrated pronounced cytotoxic effects in vitro, but the acute single-dose administration in mice was safe, suggesting acceptable short-term tolerability. Yet, with repeated administration over 28 days, marked metabolic disturbances emerged, particularly impairing hepatic and renal function. These adverse outcomes were linked to oxidative stress and lipid peroxidation, as has been reported for other polyoxovanadates compounds [21,22]. These findings underscore the necessity for caution regarding the biomedical application of redox active vanadates.

Titova and colleagues [Contribution 12] reviewed rare earth metal nanoparticles, such as those derived from gadolinium, europium, ytterbium, holmium, lutetium, dysprosium, erbium, terbium, thulium, scandium, yttrium, lanthanum, europium, neodymium, promethium, samarium, praseodymium, or cerium, which are increasingly being recognized for their potential in biomedicine. These systems provide many applications in imaging, cancer therapies, and regenerative medicine. However, the review also describes practical problems such as inconsistent toxicity profiles and the need for further modification techniques, such as surface functionalization, to make these materials safer for practical medical use.

In conclusion, optimizing the therapeutic impact of the metal nanoparticles requires a complex understanding and careful control of how the nanoparticles interact with biological systems, while minimizing potentially harmful side-effects and environmental risks. Despite the exciting progress presented in this Special Issue, several key challenges must be overcome before metal nanoparticles can be widely used in living systems [11,12,13]. Comprehensive toxicological profiling will provide information that will be very valuable. The complexity of nanoparticle interactions with biological systems demands more detailed *in vivo* studies addressing biodistribution, long-term toxicity, immunogenicity, and clearance mechanisms. Differentiating between beneficial cytotoxicity against pathogens or cancer cells and harmful side-effects affecting healthy tissues is critical [9,14], and increased specificity is paramount. Increased standardization and reproducibility are needed, possibly through understanding the nature of the inhomogeneity of the samples and the correspondingly different responses to such samples. The variability in synthesis methods, nanoparticle size, shape, and surface chemistry complicate the comparison of the biological effects across studies. Establishing standardized protocols for synthesis, characterization, and biological evaluation will be essential in order to translate the laboratory findings into clinical practice [15,16].

Targeting and functionalization strategies remain key aspects that could provide improved results in the future. The functionalization of nanoparticles with targeting ligands, biomolecules, or drugs holds great promise for enhancing their specificity and reducing their systemic toxicity. Future research focusing on the optimization of this process in order to improve targeted delivery and controlled release could be important [17,18,19,20].

Oxidative stress involves not only the generation of reactive oxygen species but also their processing. Understanding and modulating these could be a key mechanism for antimicrobial and anticancer effects. Excessive amounts of free radicals formed because of oxidative stress may induce adverse side-effects. Fine-tuning the redox activity through doping and surface modification represents a potential approach for safer nanomedicines [23,24].

Environmental considerations may affect the various applications of nanoparticles and materials, necessitating regulatory frameworks to address the unique properties of these materials, including their potential environmental effects, bioaccumulation, and disposal. The development of eco-friendly “green” synthesis methods and the evaluation of environmental toxicity will enhance the development of sustainable nanotechnology applications [25].

Finally, integration with personalized medicine is an area of future importance. The diverse biological effects of nanoparticles, depending on size, composition, and functionalization, suggest that these materials have great potential for personalized therapies that satisfy individual patient needs. Future studies should explore their integration in genomics and proteomics [13,20,26] applications. Hence, there is no doubt that the application of these materials will increase in the future.

Finally, we would like to thank the authors, reviewers, and other contributors who helped make this Special Issue a reality. We look forward to learning more about future research directions in the second edition of this Special Issue, which we expect will further unravel the potential of metal nanoparticles in advancing pharmaceutical science.
